# Comparative profiling of selected pattern recognition receptor (PRR) pathways reveals functional heterogeneity of monocytic models in the monocyte activation test

**DOI:** 10.1080/19768354.2026.2749253

**Published:** 2026-10-29

**Authors:** Ji-youn Lim, Soyoung Jeon, Yeram Jeon, Mihye Kim, Hyungjung Koh, Seoyoung Wang, Young-Joo Jeon, Jangwook Lee, Sung-Jin Yoon, Wantae Kim, Boksik Cha

**Affiliations:** a New Drug Development Center, Daegu-Gyeongbuk Medical Innovation Foundation, Daegu, Republic of Korea; b Department of Life Science, University of Seoul, Seoul, Republic of Korea; c Biopharmaceutical and Herbal Medicine Evaluation Department, National Institute of Food and Drug Safety Evaluation, Chungju, Republic of Korea; d Department of Physiology, Daegu Catholic University School of Medicine, Daegu, Republic of Korea; e Metabolic Regulation Research Center, Korea Research Institute of Bioscience and Biotechnology (KRIBB), Daejeon, Republic of Korea; f Biotherapeutics Translational Research Center, Korea Research Institute of Bioscience and Biotechnology, Daejeon, Republic of Korea; g Department of Biomolecular Science, Korea Research Institute of Bioscience and Biotechnology, School of Bioscience, Korea University of Science and Technology, Daejeon, Republic of Korea; h Environmental Disease Research Center, Korea Research Institute of Bioscience and Biotechnology, Daejeon, Republic of Korea; i Department of Functional Genomics, Korea Research Institute of Bioscience and Biotechnology, School of Bioscience, Korea University of Science and Technology, Daejeon, Republic of Korea

**Keywords:** Monocyte activation test (MAT), endotoxin, non-endotoxin pyrogens (NEPs), Toll-like receptors (TLRs)

## Abstract

The monocyte activation test (MAT) detects pyrogenic contamination from the cytokine response of human monocytes and is used as an alternative to the rabbit pyrogen test. PBMC are the reference cell source for the MAT; however, donor variability and limited supply have led to frequent substitutions with the cell lines THP-1, HL-60, and U937. Consequently, whether these lines reproduce PBMC behavior beyond that of endotoxins across receptor pathways remains unknown. In this study, we compared the IL-6 responses of PBMCs and cell lines to 10 pyrogenic stimuli acting through Toll-like receptors (TLRs) and NOD2. All four systems were assayed in parallel, and each stimulus was tested over a full dose range. Basal transcript levels of the corresponding receptors were measured using RT-qPCR in the same cells and compared with publicly available expression data. Detection limits, precision estimates, and endotoxin equivalent potencies were reported for each stimulus. PBMCs responded to every stimulus in the panel and produced the highest maximal IL-6 for most pathways; the cell lines responded only at higher ligand concentrations and over the compressed dose–response ranges. These results indicate that the relationship between primary and immortalized monocytic systems depends on the receptor engaged, rather than a fixed reduction in sensitivity. Performance against endotoxins does not predict performance against non-endotoxin pyrogens, which are the contaminants that MAT is used to detect.

## GRAPHICAL ABSTRACT

**Figure:**
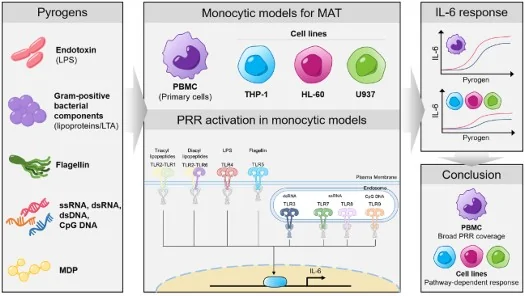

## Introduction

Pyrogenic contamination of injectable medicines, biologics and medical devices causes fever, and at sufficient dose circulatory shock (Hartung and Wendel 1995; Schindler et al. 2009; Abdellaoui et al. 2023). The rabbit pyrogen test and the Limulus amebocyte lysate (LAL) assay are two assays routinely performed for the detection of pyrogens. The rabbit pyrogen test measures the febrile response of an intact animal; thus, it registers whatever the rabbit senses and whether the contaminant has been identified. Consequently, batch release still requires animal testing; rabbits are insensitive to several non-endotoxin pyrogens (NEPs) that are pyrogenic in humans, such as lipoteichoic acids and lipopeptides of gram-positive organisms (Hartung et al. 1998; Hasiwa et al. 2013; Hartung 2015; da Silva et al. 2016). The LAL assay is sensitive to approximately 0.005 EU/mL (Hasiwa et al. 2013; Bolden et al. 2016; Neun and Dobrovolskaia 2019), but the reaction it exploits is the coagulation cascade of the horseshoe crab, which is triggered by lipopolysaccharide and by little else; contaminants from Gram-positive bacteria, fungi and viruses do not appear in the readout (Hasiwa et al. 2013; Bolden et al. 2016; Neun and Dobrovolskaia 2019).

The Monocyte Activation Test (MAT) has emerged as one of the most promising alternatives to animal-based pyrogen testing (Schindler et al. 2009; Hasiwa et al. 2013; Langezaal et al. 2002; Daneshian et al. 2009; Fernandes Silva et al. 2018; Hartung 2021; Gimenes et al. 2024), and replaces the animal with the cell type that mediates the febrile response. Human monocytes exposed to a pyrogenic sample release interleukin-6 (IL-6), tumor necrosis factor-α (TNF-α) and interleukin-1β (IL-1β) (Ahn et al. 2024), and the concentration of one of these cytokines is read against an endotoxin standard curve to report the pyrogenic load (Tindall et al. 2021; Akira and Takeda 2004; Kim et al. 2024). Monocytes carry Toll-like receptors (TLRs) and NOD-like receptors as well as the TLR4 complex, so the readout is not restricted in principle to lipopolysaccharide (Hartung 2021; Hoffmann et al. 2005; Oh et al. 2025). The method was adopted by the European Pharmacopoeia as general chapter (2.6.30), and whole-blood and cell-line formats have since been validated against the rabbit test for product classes (Schindler et al. 2009; Hasiwa et al. 2013; Langezaal et al. 2002; Daneshian et al. 2009; Fernandes Silva et al. 2018; Hartung 2021; Gimenes et al. 2024).

Which receptor is engaged depends on the class of contaminant. Lipopolysaccharide is sensed by TLR4, triacylated lipopeptides by the TLR1/2 heterodimer, diacylated lipopeptides by TLR2/6, peptidoglycan and intact Gram-positive bacteria by TLR2, flagellin by TLR5, small-molecule mimetics of single-stranded RNA by TLR7 and TLR7/8, unmethylated CpG DNA by TLR9, and muramyl dipeptide by the cytosolic receptor NOD2 (Kawai and Akira 2010; Deifl et al. 2014; Kim et al. 2025; Bakiallah et al. 2025; Chi et al. 2025). We selected one ligand for each of these pathways, so that the panel covers the receptor classes through which pyrogenic contamination is likely to be recognized. Investigators comparing MAT systems have so far worked with endotoxin, and in a few cases with one or two additional ligands (Hoffmann et al. 2005; Basketter et al. 2012; Borton and Coleman 2018). Primary peripheral blood mononuclear cells (PBMC) are the reference cell source for MAT because they carry the monocyte populations that mediate the febrile response in vivo, together with the other blood leukocytes that modulate it. However, supply limitations, such as differences in donors and lots, and availability of materials limit their use (Schindler et al. 2009; Jeon et al. 2025). As a result, immortalized monocytic cell lines such as THP-1, HL-60, and U937 are frequently used as alternative cellular models due to their experimental reproducibility and scalability (Auwerx 1991; Chanput et al. 2014; Unenkhuu et al. 2021). Previous comparisons between primary human immune cells and monocytic cell lines have focused on endotoxin or on a small number of stimuli (Schildberger et al. 2013; Bosshart and Heinzelmann 2016). Whether the relationship between the two holds across receptor pathways has not been examined. Although an endotoxin comparison would be sufficient when uniformly scaling down responsiveness in cell lines, if it were pathway-dependent, it would not suffice.

We therefore asked whether the difference between PBMC and monocytic cell lines is constant across receptor pathways or depends on which receptor is engaged. IL-6 responses of PBMC, THP-1, HL-60 and U937 were compared for ten pyrogenic stimuli acting through TLR1/2, TLR2, TLR2/6, TLR4, TLR5, TLR7, TLR7/8, TLR9 and NOD2. The four systems were assayed simultaneously, and each stimulus was evaluated over a full dose range. A constant difference appeared as a fixed rank order with parallel curves, whereas a pathway-dependent difference does not. Which of the two holds determines whether an endotoxin comparison can stand in for the rest.

## Materials and methods

### Human monocytic cell culture

Human monocytic cell lines were obtained from ATCC: THP-1 (TIB-202), HL-60 (CCL-240) and U937 (CRL-1593.2), and were used between passages 5 and 13. Cells were maintained in RPMI-1640 medium supplemented with 10% heat-inactivated fetal bovine serum (FBS) and 1% penicillin–streptomycin at 37 °C in a humidified incubator with 5% CO₂. Cells were passaged every 2-3 days and maintained in logarithmic growth phase throughout the experiments. For stimulation assays, cells were seeded at a density of 2.5 × 10⁵ cells per well in 96-well plates.

### Human peripheral blood mononuclear cells (PBMCs)

Cryopreserved PBMC from three healthy donors (BPS Bioscience, #79059) were thawed in a 37 °C water bath, washed with phosphate-buffered saline, and resuspended in RPMI-1640 supplemented with 10% fetal bovine serum. The three preparations were pooled in equal proportion before use to provide a single cell source of defined composition; this pool was used in all experiments. Pooled PBMC were seeded at 1 × 10⁵ cells per well in 96-well plates. Each concentration was assayed in five replicate wells, and each complete dose–response experiment was repeated on three independent days using separately thawed aliquots.

### Pyrogenic stimuli

Endotoxin (USP Reference Standard Endotoxin, Lonza #E700) was reconstituted in LAL reagent water and diluted to give a working range of 0.0001–10 EU/mL. All non-endotoxin pyrogens were purchased from InvivoGen and diluted serially in LAL reagent water immediately before use: Pam3CSK4 (#tlrl-pms), peptidoglycan from *Staphylococcus aureus* (PGN-SA, #tlrl-pgns2), heat-killed *S. aureus* (HKSA, #tlrl-hksa), FSL-1 (#tlrl-fsl), flagellin (#tlrl-pbsfla), imiquimod (#tlrl-imqs-1), CL075 (3M-002, #tlrl-c75), ODN2006 (CpG-B, #tlrl-2006) and muramyl dipeptide (MDP, #tlrl-mdp). The receptors involved in each process are listed in Table 1. HKSA suspensions were quantified by optical density, taking OD₆₀₀ = 1 as 10⁸ cells/mL.

**Table 1.** Analytical performance characteristics of the PBMC-based monocyte activation test (MAT) for endotoxin and non-endotoxin pyrogens.

TLR	Stimulus	Unit	LOD	LOQ	Linearity
TLR 1/2	Pam3CSK4	ng/mL	0.0135 ± 0.0008	0.0177 ± 0.0013	R^2^ > 0.99
TLR 2	HKSA	10^5^/mL	2.295 ± 0.022	4.324 ± 0.046	R^2^ > 0.99
TLR 2	PGN-SA	μg/mL	0.138 ± 0.011	0.211 ± 0.008	R^2^ > 0.98
TLR 2/6	FSL-1	μg/mL	0.011 ± 0.006	0.015 ± 0.005	R^2^ > 0.99
TLR 3	Poly I:C	μg/mL	N.D.	N.D.	Not determined
TLR 4	Endotoxin	EU/mL	0.00030 ± 0.00007	0.00050 ± 0.00007	R^2^ > 0.99
TLR 5	Flagellin	μg/mL	0.0080 ± 0.0023	0.0087 ± 0.0016	R^2^ > 0.99
TLR 7	Imiquimod	μg/mL	1.84 ± 0.14	2.71 ± 0.11	R^2^ > 0.94
TLR 7/8	CL075	μg/mL	0.0255 ± 0.0002	0.0265 ± 0.0002	R^2^ > 0.91
TLR 9	ODN2006	μg/mL	0.110 ± 0.006	0.14 ± 0.04	R^2^ > 0.92
NOD2	MDP	μg/mL	0.0267 ± 0.0012	0.0494 ± 0.0013	R^2^ > 0.99

Data are presented as mean ± SD from three independent experiments (n = 3). LOD and LOQ are expressed as the corresponding stimulus concentrations. LOD, limit of detection; LOQ, limit of quantification; R², coefficient of determination; N.D., not determined.

### Pyrogen exposure and cytokine measurement

For pyrogen stimulation assays, human monocytic cell lines (THP-1, HL-60, U937) and PBMCs were seeded in 96-well plates. Immediately after seeding, the cells were treated with each of the 10 pyrogenic stimuli in culture media containing 2% FBS for 18-20 h at 37 °C. Subsequently, IL-6 levels were measured using a human IL-6 ELISA kit (R&D Systems, D6050B) according to the manufacturer's instructions. Cytokine measurements were performed in five replicate wells at each concentration, and each complete dose–response experiment was repeated on three independent days. IL-6 concentrations were plotted against log stimulus concentration and fitted with a four-parameter logistic model in GraphPad Prism. Relative potency was obtained by interpolating, onto the endotoxin standard curve generated in the same experiment, the concentration of each non-endotoxin stimulus that produced an IL-6 response of approximately 100 pg/mL in PBMC, and is expressed in endotoxin-equivalent units (Table 3).

### Determination of analytical performance parameters

Blank samples were unstimulated wells containing cells and medium alone, measured in five replicates. The limit of detection was defined as the IL-6 concentration corresponding to the mean blank signal plus three standard deviations of the blank, and the limit of quantification as the mean blank signal plus ten standard deviations. The corresponding stimulus concentration was then obtained by back-interpolation from the fitted four-parameter logistic curve; the values reported in Table 1 are these stimulus concentrations. Intra-assay precision was determined from the replicate wells of a given condition on a single plate (n = 5). Inter-assay precision was determined from the run means of three independent experiments performed on separate days. Precision is expressed as the coefficient of variation, calculated as the standard deviation divided by the mean and multiplied by 100.

### Quantitative real-time PCR analysis

PBMCs and HL-60, THP-1, and U937 cells were cultured and then harvested, and total RNA was isolated using a QIAGEN RNA isolation kit (QIAGEN, Germany) according to the manufacturer’s instructions, including genomic DNA removal. cDNA was synthesized from the purified total RNA using ReverTra Ace™ qPCR RT Master Mix (TOYOBO Co., Ltd., Japan) according to the manufacturer’s protocol. qPCR was performed using gene-specific primer pairs and SYBR™ Green Universal Master Mix (Thermo Fisher Scientific, USA) on a CFX96 Dx Real-Time PCR Detection System (Bio-Rad Laboratories, USA). For each target gene, the mean expression level in PBMCs was set to 1, and expression levels in HL-60, THP-1, and U937 cells were expressed as fold changes relative to PBMCs. Primer sequences are provided in Supplementary Table S1.

### Basal pattern recognition receptor (PRR) expression analysis

Normalized transcript per million (nTPM) values for the TLRs and NOD2 examined in this study were retrieved from the Human Protein Atlas (https://www.proteinatlas.org). Values for THP-1, HL-60 and U937 were taken from the cell Line RNA dataset, and values for PBMC from total PBMC entry of the immune cell dataset. nTPM values represent transcript abundance normalized across samples as defined by the Human Protein Atlas. These data derive from independent samples rather than from the cell preparations used in the functional experiments.

### Statistical analysis

All data are presented as mean ± standard deviation (SD). Statistical analyzes were performed using GraphPad Prism and R software. Dose–response curves were analyzed using nonlinear regression where appropriate. Transcript levels in each cell line were compared with PBMC by unpaired two-tailed Student's t-test. Statistical significance was set at *p* < 0.05.

## Results

### Analytical performance of the PBMC-based monocyte activation test

We first characterized the PBMC assay for each stimulus in the panel: ligands for TLR1/2, TLR2, TLR2/6, TLR3, TLR4, TLR5, TLR7, TLR7/8, TLR9 and the cytosolic receptor NOD2. Limits of detection and quantification, the units in which each is expressed, and the linearity of the fitted curve are given in Table 1. Endotoxin was detected at 0.0003 ± 0.00007 EU/mL and quantified at 0.0005 ± 0.00007 EU/mL, the lowest values in the panel. Among the non-endotoxin stimuli the limits of quantification spanned three orders of magnitude, from 0.0087 ± 0.0016 µg/mL for flagellin and 0.0151 ± 0.0049 µg/mL for FSL-1–2.7055 ± 0.1143 µg/mL for imiquimod. Pam3CSK4 was quantified at 0.0177 ± 0.00134 ng/mL, three orders of magnitude below the other lipopeptide FSL-1. Linearity exceeded r² = 0.98 for six of the nine stimuli that showed a fitted curve, and was lowest for CL075 (r² > 0.91), ODN2006 (r² > 0.92) and imiquimod (r² > 0.94). Of the nine remaining stimuli, imiquimod was the only one whose curve did not reach a plateau within the concentration range tested, and its limit of quantification was 150-fold higher than that of the next least sensitive stimulus. The remaining fitted curves plateau within the tested range.

### Precision and reproducibility of MAT responses

Intra-assay CVs ranged from 7.4 to 28.0% and inter-assay CVs from 0.1 to 31.8% across the 10 stimuli evaluated (Table 2). For most stimuli, inter-assay variation was lower than intra-assay variation. The two were calculated on different bases: intra-assay CVs from replicate wells within a single plate, inter-assay CVs from the mean values of three independent experiments. Pooling PBMC before use also removes donor as a source of between-run variation.

**Table 2.** Intra- and inter-assay precision of IL-6 responses in the PBMC-based MAT.

	Intra-Assay CV(%)			Inter-Assay CV(%)		
Pyrogen	0.2x Dilution	1x Dilution	5x Dilution	0.2x Dilution	1x Dilution	5x Dilution
Pam3CSK4	12.6	7.5	7.4	3.8	4.0	4.7
FSL-1	13.9	15.7	26.2	6.4	16.7	31.8
Endotoxin	10.9	12.3	11.2	10.2	4.9	9.5
Flagellin	16.9	12.8	13.3	1.1	5.9	0.1
Pyrogen	0.3x Dilution	1x Dilution	3x Dilution	0.3x Dilution	1x Dilution	3x Dilution
HKSA	17.5	13.1	15.4	4.3	1.6	2.5
MDP	28.0	8.3	7.0	16.2	3.5	7.0
PGN-SA	20.1	12.3	8.0	1.2	3.0	3.4
Imiquimod	16.5	14.5	8.7	1.5	4.6	4.2
ODN2006	9.1	10.3	8.6	1.0	1.9	2.2

Intra-assay precision was calculated from replicate measurements within a single experiment, whereas inter-assay precision was calculated across independent experiments. Data are presented as coefficient of variation (CV, %). Depending on the working concentration range of each pyrogen, either 0.2×/1×/5× or 0.3×/1×/3× dilution levels were used.

### Endotoxin responsiveness reveals strong TLR4 signaling capacity in PBMCs

IL-6 production was first measured after stimulation with lipopolysaccharide (LPS), the canonical TLR4 ligand. PBMCs responded well and exhibited the highest IL-6 levels among the four systems (Figure 1(A)). HL-60 required roughly three orders of magnitude more endotoxin to respond and did not reach a plateau within the range tested, so that its response remained log-linear up to 20 EU/mL (Figure 1(B)). U937 responded over the same concentration range but reached only 70 pg/mL (Figure 1(D)), and THP-1 showed little response throughout (Figure 1(C)). The combined dose–response curves illustrate these differences more clearly (Figure 1(E)). The PBMC curve was left-shifted and covered a wider response range, whereas the curves for the monocytic cell lines were narrower and shifted toward higher LPS concentrations. TLR4 transcript was below the limit of detection in all three cell lines and readily quantified in PBMC (*p* < 0.0001, Figure 1(F)), and the public dataset showed the same ordering (Figure 1(G)). HL-60 nevertheless responded to endotoxin without detectable TLR4 transcript, placing the difference between the systems downstream of basal receptor abundance.

**Figure 1.**
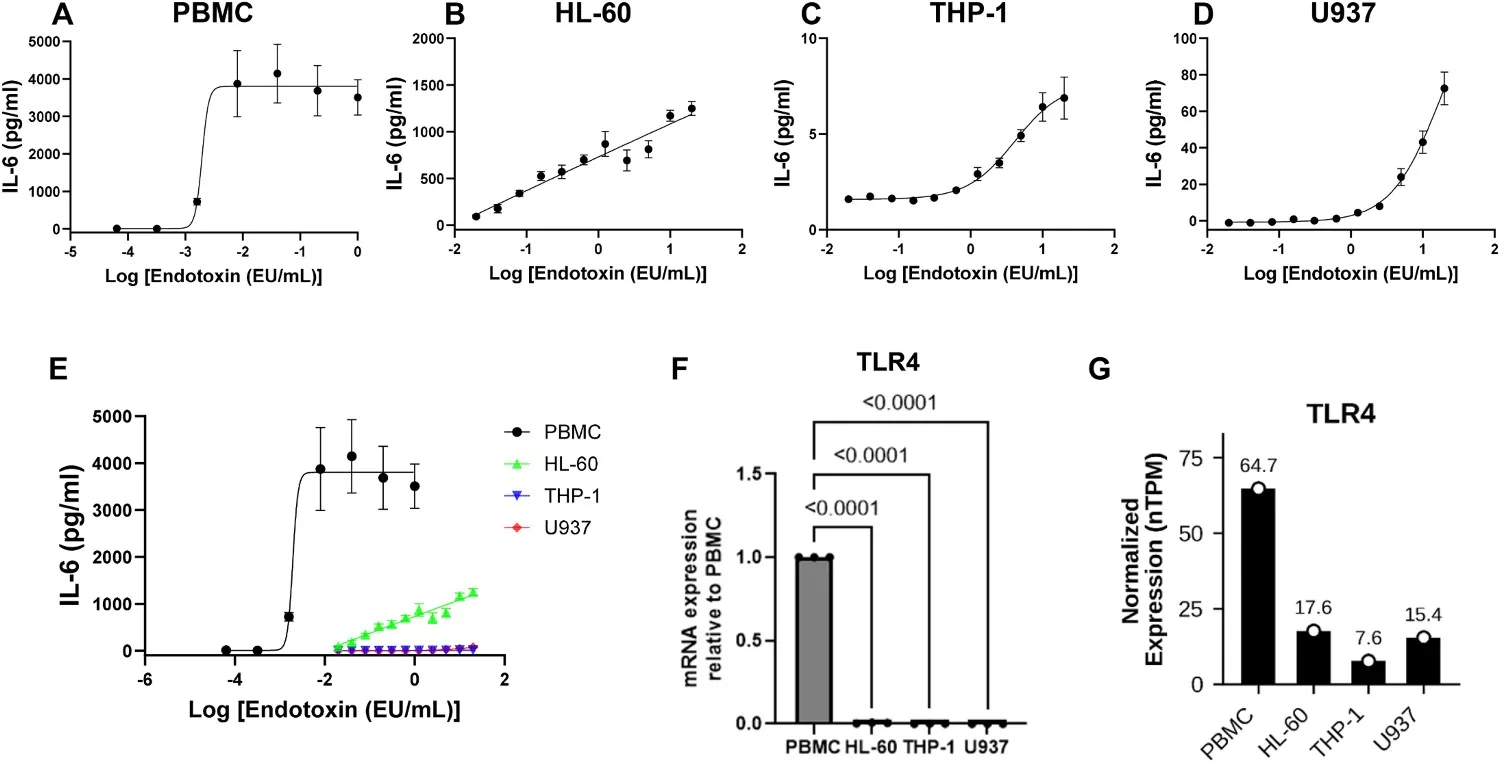
Differential endotoxin responsiveness and TLR4 expression in PBMCs and monocytic cell lines. (A–D) IL-6 release after 18 h stimulation with endotoxin in PBMC (A), HL-60 (B), THP-1 (C), and U937 (D). Note the different y-axis scales. Each concentration was assayed in five replicate wells, and each dose–response experiment was performed on three independent days; points are mean ± SD. (E) The four dose–response curves plotted on a common axis. (F) TLR4 transcript measured by RT-qPCR in the same cell preparations, normalized to *RPS18* and expressed relative to PBMC. Bars are mean ± SD of three independent experiments. *p* values are from unpaired two-tailed Student's *t*-tests against PBMC. (G) TLR4 transcript in the same four systems from the Human Protein Atlas, shown as normalized transcript per million (nTPM).

### Evaluation of non-endotoxin pyrogen responses: TLR1/2 signaling

We next examined responses to NEPs, which include structurally diverse microbial components recognized through PRRs other than TLR4 (Basketter et al. 2012). Unlike endotoxin, many NEPs are not detected by conventional LAL assays, making their detection an important consideration in MAT-based testing.

TLR1/2 responsiveness was assessed using Pam3CSK4, a synthetic triacylated lipopeptide that mimics bacterial lipoproteins and activates the TLR1/2 heterodimer. In PBMC, IL-6 production increased progressively with Pam3CSK4 concentration and reached 2,100 pg/mL (Figure 2(A)). THP-1, HL-60 and U937 produced much lower IL-6 even though they were tested over a concentration range 1,000-fold higher than that used for PBMC (Figures 2(B-D); note the difference in units), and none exceeded 180 pg/mL. The combined dose–response curves illustrate the difference (Figure 2(E)). TLR1 transcript was 3- to 17-fold lower in the three lines than in PBMC (Figure 2(F)), and the public dataset showed the same ordering (Figure 2(G)).

**Figure 2.**
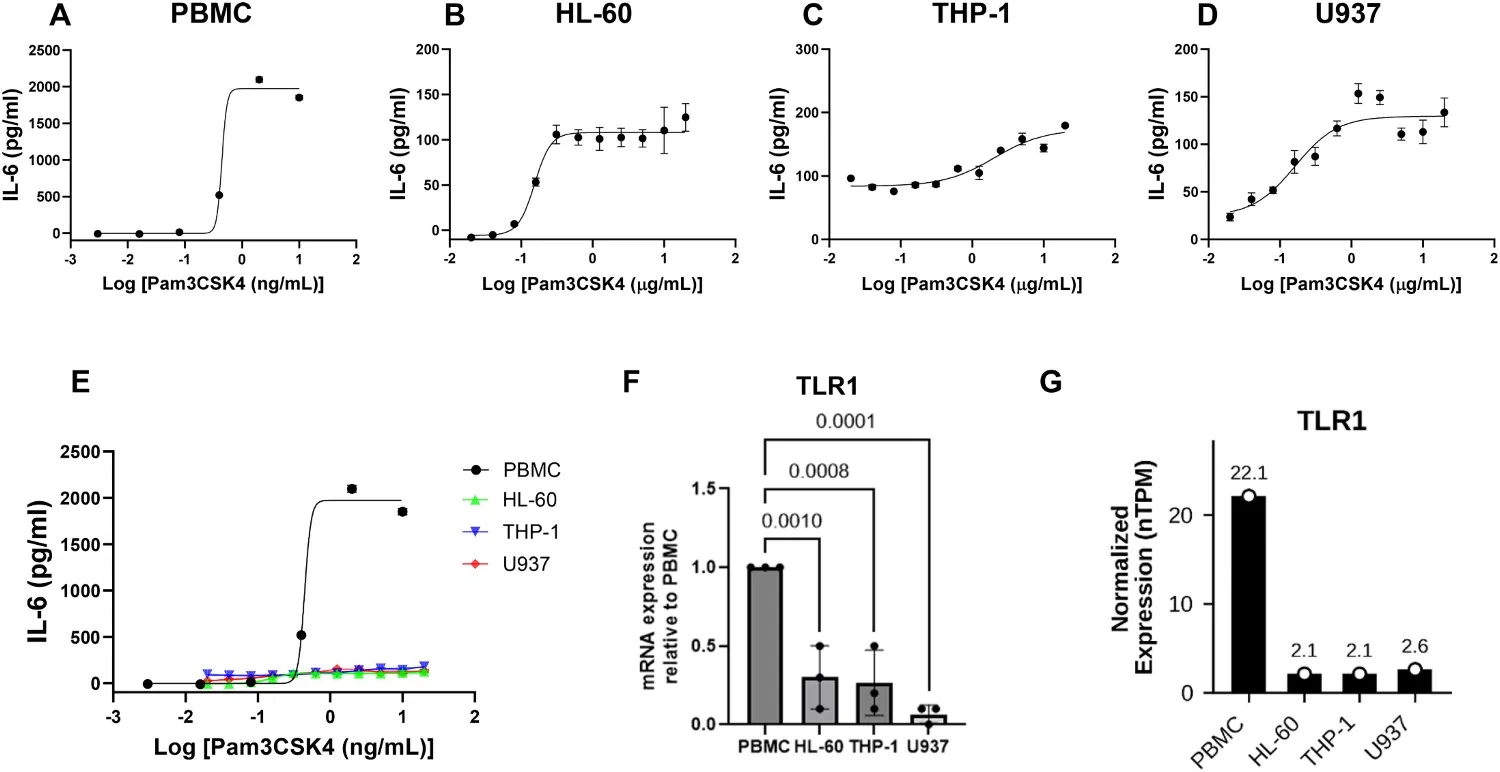
Attenuated TLR1/2-mediated responses in monocytic cell lines. (A–D) IL-6 production following stimulation with the TLR1/2 agonist Pam3CSK4 in PBMCs (A), HL-60 (B), THP-1 (C), and U937 (D). Note the different x- and y-axis scales. Each concentration was assayed in five replicate wells, and each dose–response experiment was performed on three independent days; points are mean ± SD. (E) The four dose–response curves plotted on a common axis. (F) *TLR1* mRNA levels were quantified by RT-qPCR, normalized to *RPS18*, and expressed as fold changes relative to the mean level in PBMCs. Bars are mean ± SD of three independent experiments. *p* values are from unpaired two-tailed Student's *t*-tests against PBMC. (G) TLR1 transcript in the same four systems from the Human Protein Atlas, shown as normalized transcript per million (nTPM).

### TLR2-mediated recognition of bacterial cell wall components

We next compared IL-6 responses to two Gram-positive bacterial stimuli: PGN-SA, associated with TLR2-related recognition, and HKSA, a complex whole-cell preparation. PBMC showed concentration-dependent increases in IL-6 after treatment with either stimulus (Figures 3(A and E)). THP-1, HL-60 and U937 cells responded to higher concentrations and over narrower ranges (Figures 3(B-D and F-H)). For PGN-SA the ordering was the same as that for Pam3CSK4, with PBMC reaching 1,900 pg/mL and none of the lines exceeding 240 pg/mL (Figure 3(I)). HKSA was the exception: HL-60 reached 670 pg/mL against 230 pg/mL for PBMC, whereas THP-1 and U937 remained below 50 pg/mL (Figure 3(J)). TLR2 transcript was 7- to 12-fold lower in the three lines than in PBMC (Figure 3(K)). Both datasets placed PBMC highest, although they differed in the relative position of THP-1 and HL-60 (Figures 3(K, L)).

**Figure 3.**
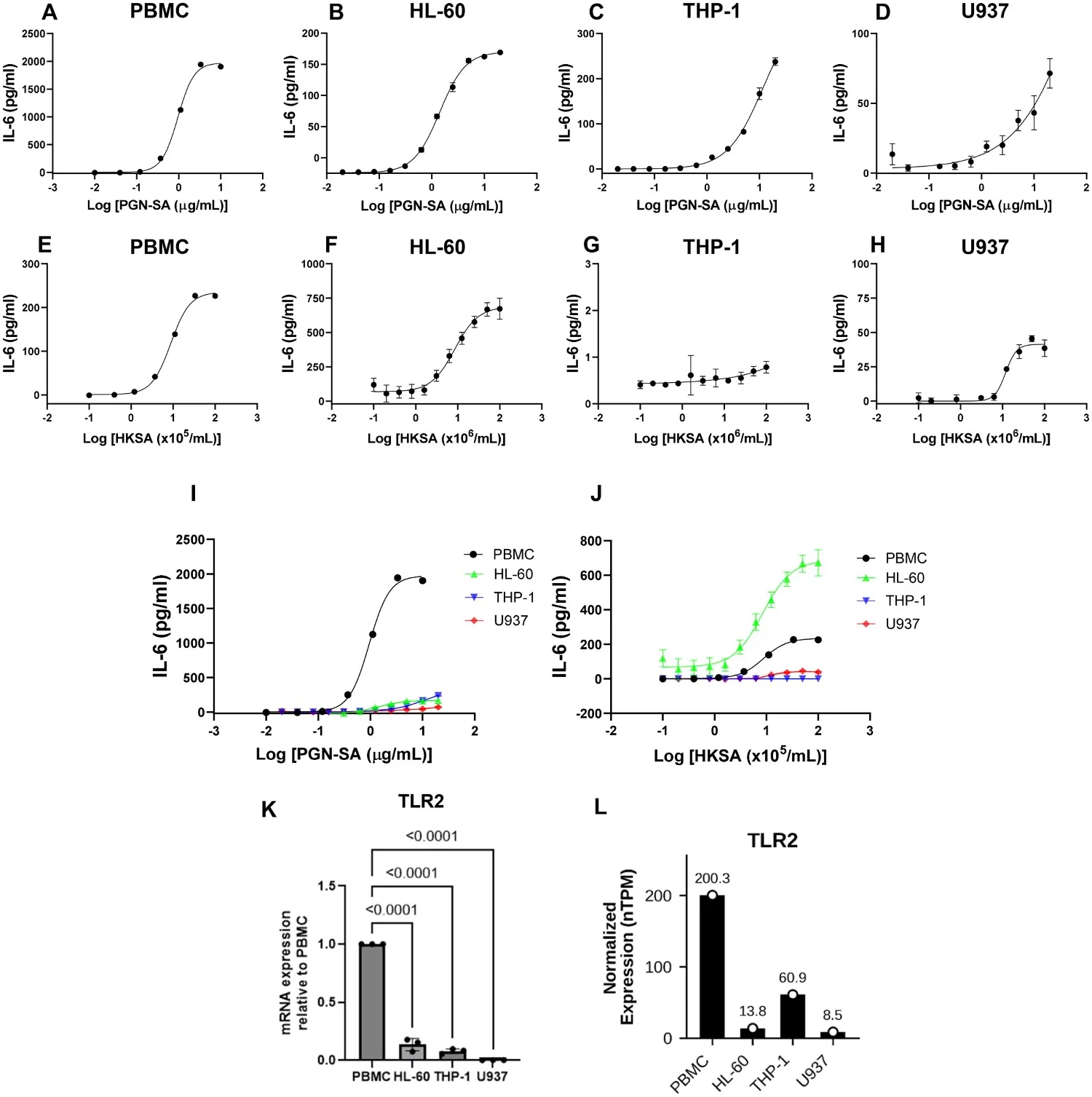
Responses to Gram-positive bacterial cell-wall stimuli in PBMC and monocytic cell lines. (A–D) IL-6 release after stimulation with PGN-SA in PBMC (A), HL-60 (B), THP-1 (C), and U937 (D). (E–H) IL-6 release after stimulation with heat-killed *Staphylococcus aureus* (HKSA) in the same cell types. Note the different y-axis scales; maximal responses span two orders of magnitude between panels. Each concentration was assayed in five replicate wells, and each dose–response experiment was performed on three independent days; points are mean ± SD. (I, J) The four dose–response curves for PGN-SA (I) and HKSA (J) plotted on common axes. (K) TLR2 transcript measured by RT-qPCR in the same cell preparations, normalized to *RPS18* and expressed relative to PBMC. Bars are mean ± SD of three independent experiments. *p* values are from unpaired two-tailed Student's *t*-tests against PBMC. (L) TLR2 transcript in the same four systems from the Human Protein Atlas, shown as normalized transcript per million (nTPM).

### IL-6 responses to FSL-1 and flagellin

We next examined responses to FSL-1, an agonist of the TLR2/6 heterodimer, and flagellin, a ligand of TLR5. FSL-1 induced a concentration-dependent IL-6 response in PBMC, reaching 1,800 pg/mL (Figure 4(A)). THP-1, HL-60 and U937 responded mainly at higher concentrations and none exceeded 450 pg/mL (Figures 4(B-E)). TLR6 transcript was lower in three cell lines than in PBMC (Figure 4(F)). The public dataset placed all three lines well below PBMC (Figure 4(G)). Flagellin produced a similar difference among the cellular models. PBMC reached 3,500 pg/mL (Figure 4(H)), HL-60 290 pg/mL (Figure 4(I)), and THP-1 and U937 remained below 5 pg/mL over the tested range (Figures 4(J-L)). TLR5 transcript was below the limit of detection in all three lines (Figure 4(M)), and the public dataset gave the same result (Figure 4(N)). HL-60 nevertheless responded to flagellin at the higher concentrations tested (Figure 4(I)), so that basal transcript level did not by itself predict whether a system would respond, as was also the case for endotoxin.

**Figure 4.**
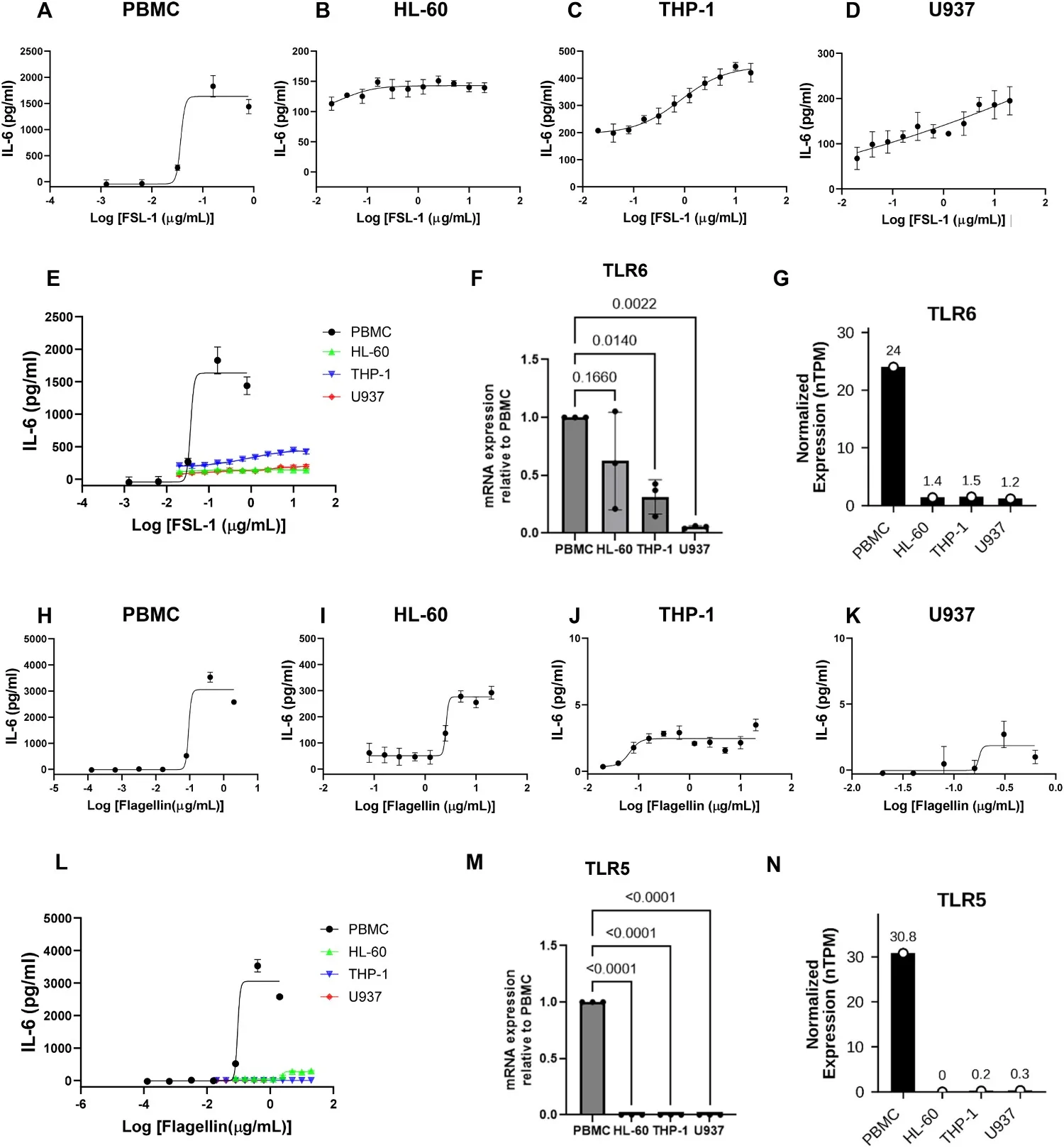
TLR2/6 and TLR5 responses across the four monocytic systems. (A–D) IL-6 release after 18 h stimulation with the TLR2/6 agonist FSL-1 in PBMC (A), HL-60 (B), THP-1 (C), and U937 (D). (E) FSL-1 dose–response curves for the four systems on a common axis. (F) TLR6 transcript measured by RT-qPCR in the same cell preparations, normalized to *RPS18* and expressed relative to PBMC. (G) TLR6 transcript from the Human Protein Atlas, shown as normalized transcript per million (nTPM). (H–K) IL-6 release after stimulation with the TLR5 agonist flagellin in the same four systems, and (L) the corresponding overlay. (M, N) TLR5 transcript by RT-qPCR (M) and from the Human Protein Atlas (N). Note the different y-axis scales throughout; maximal responses differ by up to three orders of magnitude between panels. Each concentration was assayed in five replicate wells, and each dose–response experiment was performed on three independent days; points are mean ± SD. Bars in (F), (G), (M) and (N) are mean ± SD of three independent experiments, and *p* values are from unpaired two-tailed Student's *t*-tests against PBMC. Human Protein Atlas values derive from independent samples and not from the cells used here.

### Different response patterns to TLR7 and TLR7/8 stimulation

We examined cellular responses to the selective TLR7 agonist, imiquimod, and the dual TLR7/8 agonist, CL075, to investigate the endosomal RNA-sensing pathways involved in NEP detection. Imiquimod increased IL-6 production in PBMC in a concentration-dependent manner, reaching 570 pg/mL (Figure 5(A)), whereas THP-1 and HL-60 produced no detectable IL-6 at any concentration tested (Figures 5(B, C, H)). The response to CL075 was less uniform. PBMC remained the most responsive model at 3,000 pg/mL, HL-60 and U937 produced intermediate amounts of 1,400 and 650 pg/mL, and THP-1 responded only at the highest concentration tested (Figures 5(D-G, I)). TLR7 and TLR8 transcripts were 4- to 100-fold lower in the three lines than in PBMC (*p* < 0.0001 for all comparisons, Figures 5(J, K)), and the public dataset gave the same result (Figures 5(L, M)). The transcript levels did not distinguish the two agonists: HL-60 carried the same low TLR7 and TLR8 transcript levels whether it was exposed to imiquimod, to which it did not respond, or to CL075, to which it responded with 1,400 pg/mL.

**Figure 5.**
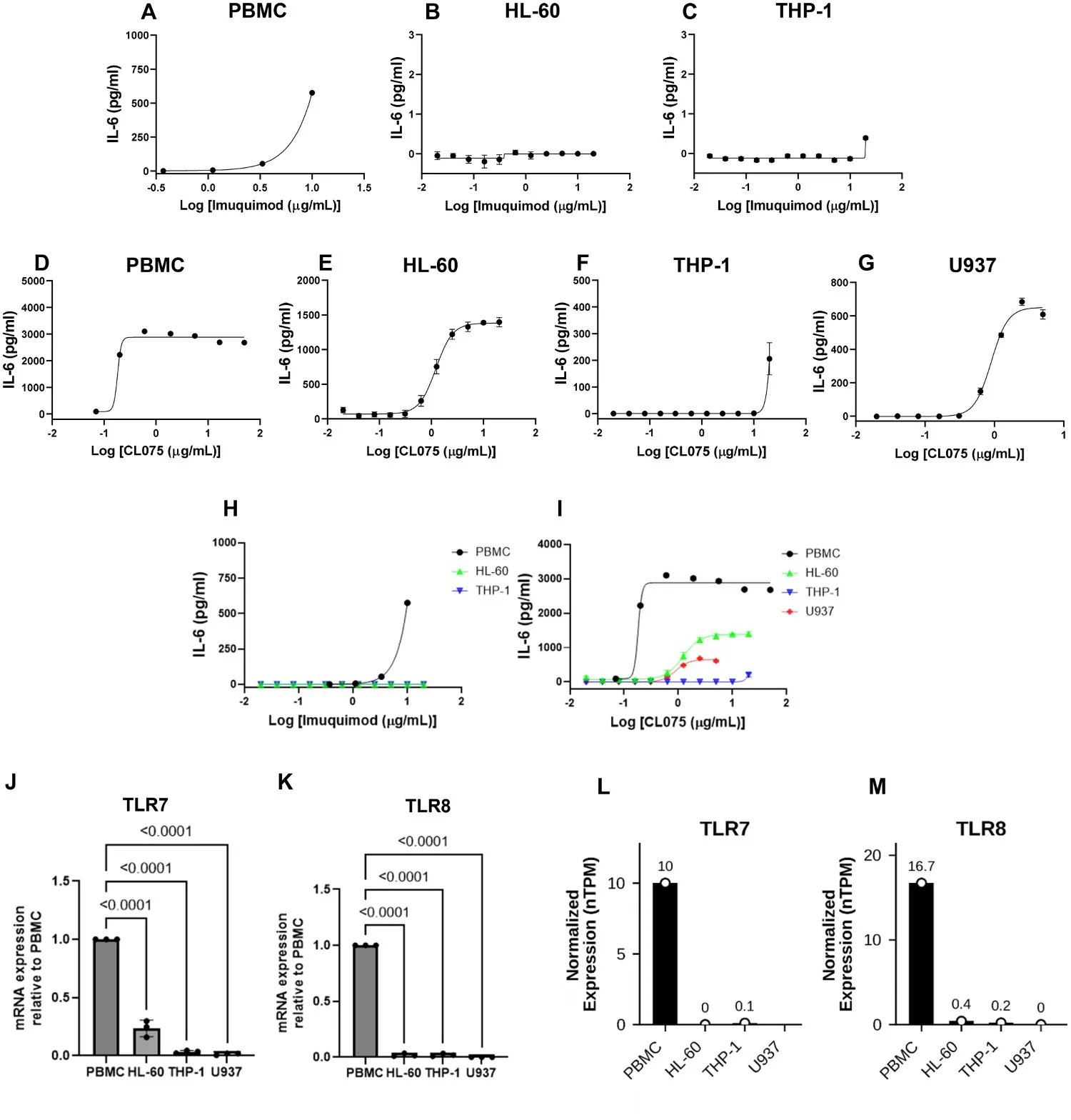
Divergent responses to TLR7-selective and TLR7/8 dual agonists. (A–C) IL-6 release after stimulation with imiquimod in PBMC (A), HL-60 (B), and THP-1 (C). (D–G) IL-6 release after stimulation with CL075 in PBMC (D), HL-60 (E), THP-1 (F), and U937 (G). (H, I) Overlay comparisons for imiquimod (H) and CL075 (I). (J–M) TLR7 and TLR8 transcript by RT-qPCR (J, K) and from the Human Protein Atlas (L, M). Imiquimod produced no measurable IL-6 in either cell line tested, whereas all three lines responded to CL075. Assay and statistical details are as in Figure 4.

### Distinct activation pattern of the TLR9 pathway across monocytic systems

To evaluate innate immune responses mediated by endosomal DNA-sensing pathways, we examined responses to the TLR9 agonist ODN2006. ODN2006 produced a response pattern that differed from those observed with the other stimuli. PBMC showed a concentration-dependent increase in IL-6 production and plateaued at 40 pg/mL (Figure 6(A)), whereas HL-60 continued to rise across the range tested and reached 57 pg/mL at the highest concentration, exceeding PBMC (Figure 6(B)). THP-1 and U937 showed little response throughout (Figures 6(C, D)). The difference among the four models is evident in the combined dose–response plot (Figure 6(E)). HL-60 carried 2.9-fold more TLR9 transcript than PBMC, whereas THP-1 and U937 did not differ significantly from PBMC (Figure 6(F)); the public dataset likewise placed HL-60 highest (Figure 6(G)). This is the only receptor in the panel for which a cell line exceeded PBMC, and ODN2006 the only stimulus for which a cell line gave the larger IL-6 response.

**Figure 6.**
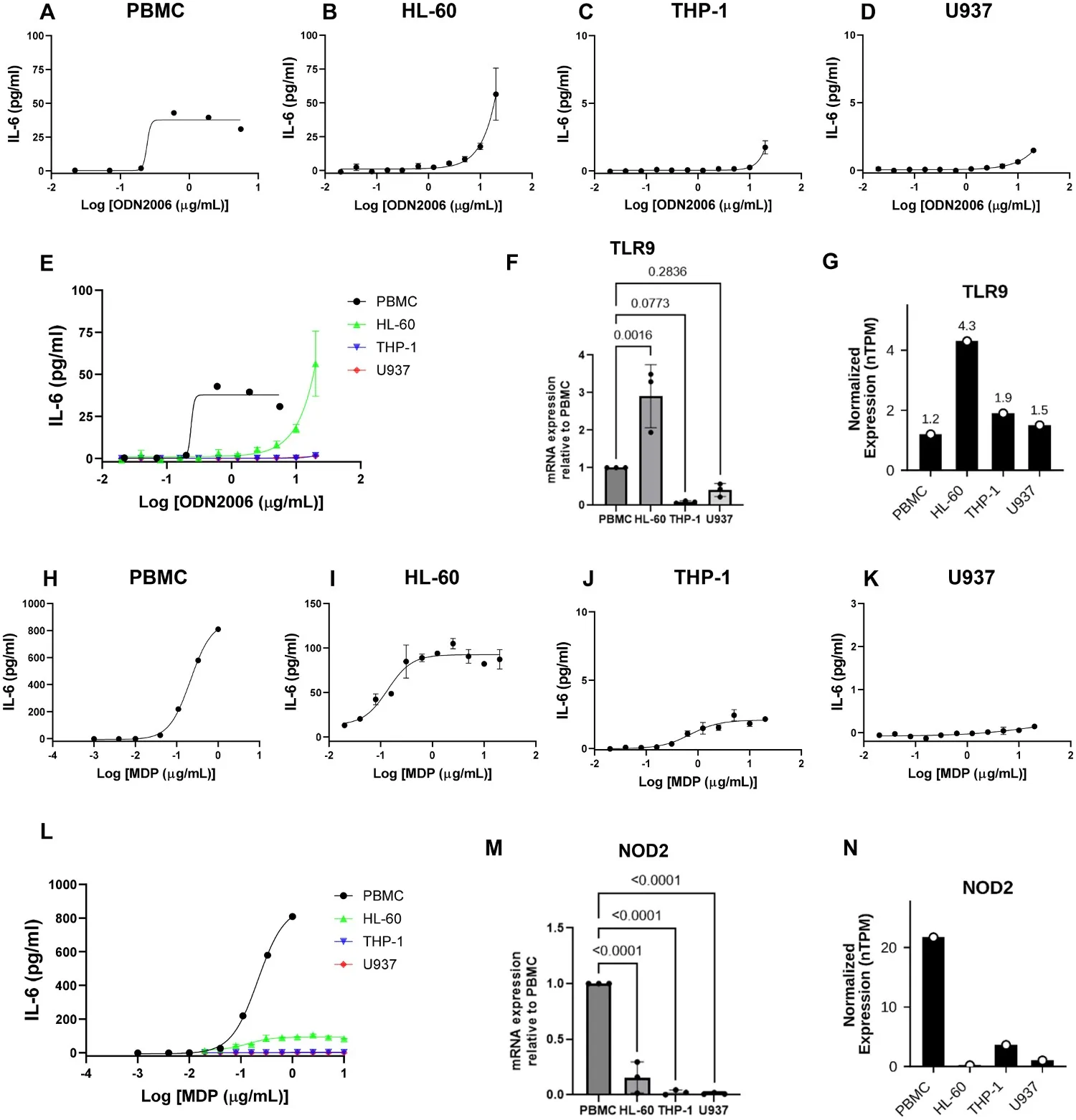
Distinct activation of TLR9 and attenuation of NOD2 signaling across monocytic systems. (A–D) IL-6 release after stimulation with the TLR9 agonist ODN2006 in PBMC (A), HL-60 (B), THP-1 (C), and U937 (D), and (E) the corresponding overlay. (F, G) TLR9 transcript by RT-qPCR (F) and from the Human Protein Atlas (G). HL-60 carried the highest level in both datasets. (H–K) IL-6 release after stimulation with MDP in the same four systems, and (L) the corresponding overlay. (M, N) NOD2 transcript by RT-qPCR (M) and from the Human Protein Atlas (N). Assay and statistical details are as in Figure 4.

### Differential NOD2-mediated responses to MDP in PBMCs and monocytic cell lines

We also tested muramyl dipeptide (MDP), a ligand of the intracellular receptor NOD2. PBMC showed a concentration-dependent increase in IL-6 production and reached 810 pg/mL (Figure 6(H)). HL-60 plateaued at 100 pg/mL, and THP-1 and U937 remained below 3 pg/mL over the same concentration range (Figures 6(I-K, L)). NOD2 transcript was 6- to 100-fold lower in the three lines than in PBMC (Figure 6(M)), in agreement with the public dataset (Figure 6(N)). MDP therefore followed the pattern seen for the other stimuli, with the ordering of the four systems preserved from receptor transcript through to IL-6 output. Representative IL-6 responses and relative potency estimate for endotoxin and non-endotoxin pyrogens are summarized in Table 3.

**Table 3.** Representative IL-6 responses and relative potency estimates for endotoxin and non-endotoxin pyrogens in PBMC-based MAT.

Pyrogen	Conc.	IL-6 response (pg/mL)	Relative potency (EEU/mL)
Endotoxin (RSE)	0.0012 EU/mL	105 ± 11	1.0
Pam3CSK4	0.3 ng/mL	107 ± 13	0.0036
FSL-1	0.03 μg/mL	101 ± 14	0.0040
Flagellin	0.07 μg/mL	101 ± 17	0.018
CL075	0.11 μg/mL	100.6 ± 1.2	0.011
HKSA	7.5 × 105/mL	100.4 ± 1.2	0.00020
MDP	0.1 μg/mL	100.7 ± 1.7	0.018
PGN-SA	0.15 μg/mL	100.1 ± 2.3	0.0046
Imiquimod	4.4 μg/mL	100.0 ± 1.1	0.00030

Data are presented as mean ± SD from three independent experiments (n = 3). Relative potency was estimated by interpolating the IL-6 response of each non-endotoxin pyrogen onto the reference endotoxin standard curve generated in the same experiment and is expressed as endotoxin-equivalent units (EEU/mL). RSE, reference standard endotoxin.

## Discussion

Across the panel, PBMC responded to nine of the ten stimuli and gave the highest maximal IL-6 for seven of them. The difference between primary and immortalized systems was not a constant offset: its magnitude varied by pathway, and its direction reversed twice, both times in HL-60, which exceeded PBMC with HKSA and with ODN2006.

HL-60 responded to ODN2006 more strongly than PBMC at the higher ligand concentrations, one of two instances in the panel in which a cell line exceeded PBMC, the other being HKSA. The three cell lines are arrested at different points of myeloid development: HL-60 at the promyelocytic stage, U937 as a monoblastic line, and THP-1 as a monocytic line. In human peripheral blood, TLR9 expression is largely restricted to plasmacytoid dendritic cells and B cells and is low in mature monocytes (Unenkhuu et al. 2021). A line arrested before monocytic commitment would therefore not be expected to share the low TLR9 expression of the more committed lines, and HL-60 indeed carried 2.9-fold more TLR9 transcript than PBMC in our own measurement (*p* = 0.0016, Figure 6(F)). Transcript abundance is unlikely to be the whole account. Functional CpG recognition also requires oligonucleotide uptake, UNC93B1-dependent trafficking of TLR9 from the endoplasmic reticulum to endolysosomes, and proteolytic cleavage of the receptor in the acidified endosome; a difference at any of these steps could amplify a modest expression difference. We did not measure them. Measuring UNC93B1 expression, blocking endosomal acidification, and quantifying CpG uptake would discriminate between these possibilities. In contrast, the same explanation does not extend to HKSA, for which HL-60 exhibited better responsiveness than PBMCs while carrying seven-fold less TLR2 transcript; the difference must lie elsewhere than in receptor abundance, and requires further evaluation.

HL-60 was the weakest of the three lines for both lipopeptide agonists, reaching 110 pg/mL with Pam3CSK4 and 150 pg/mL with FSL-1, yet it was the strongest for endotoxin among the lines and the only system to exceed PBMC, which it did with both HKSA and ODN2006. A model that under-reports most pathways can therefore still be the better choice for one of them. This was the result of the panel being constructed. Endotoxin behavior in MAT systems is well characterized, and non-endotoxin behavior is not: the published comparisons cover one or two ligands at a time (Solati et al. 2022), which is enough to rank two systems for that ligand but not enough to predict how the ranking changes when the contaminant changes. Running ten stimuli on four systems in parallel and on the same days makes that comparison possible, and it shows that the ranking does change. The relative potencies in Table 3 span two orders of magnitude, from 0.018 EEU/mL for MDP to 0.0002 EEU/mL for HKSA, and the ordering of the four systems was not preserved across that span. The immediate use is negative: a sensitivity factor derived from endotoxin does not transfer to other contaminants, and the direction of the error is not predictable from the factor itself.

PBMC and the monocytic cell lines differ in more than one respect at the same time. PBMC contain monocytes as a minority population alongside lymphocytes, natural killer cells and dendritic cells, and IL-6 output reflects paracrine amplification that monoculture cannot reproduce; the lines are additionally transformed and were used undifferentiated. The present design does not separate these contributions, and isolated CD14-positive monocytes would be required to do so. Cellular composition nonetheless does not account for the whole pattern. It fits the TLR7 result well, since TLR7 is expressed mainly by plasmacytoid dendritic cells and B cells in human blood (Unenkhuu et al. 2021) and only PBMC produced measurable IL-6 in response to imiquimod. It does not fit TLR9 or HKSA, for both of which HL-60, a single myeloid population containing neither cell type, exceeded PBMC. The differences we report therefore reflect both the composition of the test system and properties of the cells themselves, in proportions that vary by pathway. We studied the cell lines in the undifferentiated state and have no data on how differentiation would change these conclusions.

Several limitations of the present study should be acknowledged. First, IL-6 production was used as the primary cytokine readout for MAT responses. Although IL-6 is widely accepted as a key biomarker in MAT assays, different PRR pathways can induce distinct cytokine profiles, including interferons and other pro-inflammatory mediators. Future studies incorporating multiplex cytokine analysis may therefore provide a more comprehensive characterization of pathway-specific immune responses. Second, the present work focused on defined PRR ligands representing model endotoxin and non-endotoxin pyrogens. Additional studies using complex biological products or contaminated pharmaceutical materials would further clarify how these response patterns translate to real-world pyrogen detection scenarios. Third, PBMC and the cell lines were assayed at different seeding densities, so the absolute IL-6 concentrations in Figures 1–6 derive from wells containing unequal cell numbers. The difference runs opposite to our conclusion: the cell lines were seeded 2.5-fold more densely and still produced less IL-6, so per-cell responsiveness differs more than the absolute values indicate. Fourth, the receptor data describe transcript and not functional surface receptor. Transcript level did not predict response magnitude: HL-60 responded to endotoxin, flagellin and CL075 while carrying little or no detectable transcript for TLR4, TLR5, TLR7 or TLR8. The concordance we report for TLR9 is therefore an association, and establishing a receptor-level mechanism would require measurement of surface receptor and of proximal signaling downstream of ligand binding. In the cell lines, several stimuli that did not induce measurable IL-6 nevertheless elicited IL-1β or TNF-α, which places the limitation in IL-6 output rather than at receptor engagement, although the step involved was not identified.

In conclusion, responsiveness to endotoxin did not predict responsiveness to non-endotoxin pyrogens. HL-60 was the strongest of the three lines for endotoxin and the weakest for both lipopeptide agonists, and it was the only system to exceed PBMC, which it did with HKSA and with ODN2006. A cell line characterized against endotoxin alone therefore carries an unknown error, of unknown sign, for every other contaminant class. Since detection of non-endotoxin pyrogens is a principal reason for adopting MAT, a cell line should be qualified against the receptor pathways relevant to the contamination a given product can plausibly carry. PBMC responded to nine of the ten stimuli and are the broader option where that profile is undefined. In contrast, cell lines retained the advantages that motivate their use: defined composition, unlimited supply, and freedom from donor variation, which the present data neither address nor call into question.

## Supplementary Material

Supplemental Material

## Data Availability

The data that support the findings of this study are available from the corresponding author upon reasonable request.
